# Linking IL-6 and hsCRP among Indian patients with myocardial infarction

**DOI:** 10.6026/973206300200378

**Published:** 2024-04-30

**Authors:** Virendra Verma, Pavan Kumar Sharma, Shashi Prabha Singh, Devajit Sarmah, Rajni Patel, Poonam Verma, Shiv Shanker Tripathi, Deepa Arya, Manish Kumar Verma

**Affiliations:** 1 Department of Medicine, Rajarshi Dashrath Autonomous State Medical College, Ayodhya, India; 2 Department of Biochemistry, Rama Medical College Hospital & Research Centre, Kanpur, India; 3 Department of Biochemistry, Maa Vindhyavasini Autonomous State Medical College, Mirzapur, India; 4 Department of Biochemistry, Rajarshi Dashrath Autonomous State Medical College, Ayodhya, India; 5 Department of Anatomy, Autonomous State Medical College, Etah, India; 6 President, Society for Scientific Research, Barabanki, India; 7 Department of Emergency Medicine, Dr. Ram Manohar Lohia Institute of Medical Sciences, Lucknow, India

**Keywords:** Myocardial infarction, Acute Coronary Syndrome, Biochemical Marker, high sensitivity C- reactive protein, Interleukin-6

## Abstract

The association between serum interleukin-6 (IL-6) and highly sensitive C - reactive protein (hsCRP) as predictors of the risk factors for Myocardial Infarction. The study included a total of 50 patients with Myocardial Infarction, aged between 25 to 74 years. The levels of hsCRP were measured using the immunoturbidimetry method, while Interleukin 6 was estimated using the sandwich ELISA method. Statistical analysis was conducted using SPSS version 21.0, with p values calculated using Quartile ratio, ANOVA unpaired t-test, and Kaplan-Meier Curve Method. A p-value of less than 0.05 was considered statistically significant. All participants underwent a questionnaire, physical examination, medical history assessment, and laboratory tests. The results of the study showed that there was a significant correlation between IL-6 and hsCRP levels in the Quartile groups, as well as with lipid profiles. The Kaplan-Meier method also demonstrated a significant association between IL-6 and hsCRP levels in participants. The comparison of biomarkers further supported these findings. Thus, data shows that elevated levels of hsCRP and IL-6 could serve as valuable diagnostic markers for predicting Acute Myocardial Infarction. Our study strongly suggests that IL-6 could be a powerful marker in evaluating the Myocardial Infarction.

## Background:

Myocardial infarction (MI), is a severe health issue that occurs when the heart's blood supply is obstructed, resulting in damage to the heart muscle [1, 2, 3]. The occurrence and mortality rates of MI are strongly linked to higher risks of death and health complications. Most studies have focused on early biomarker levels in the first 24 to 72 hours, neglecting the significance of repeated measurements in predicting long-term risks for patients with certain heart conditions. Research has shown that both men and women display changes in biochemical markers, with an increase of 30% to 50% in the general population [4, 5, 6]. Inflammatory biomarkers like IL-6 have been connected to cardiovascular events [7], supporting the theory of inflammation's role. The correlation between markers like IL-6 and a high-sensitivity C reactive protein (hs-CRP) with cardiovascular events has been established in both healthy individuals and those with illnesses [8- 9]. HS-CRP has been associated with various chronic diseases such as hypertension, metabolic syndrome, and heart disease. Inflammation plays a crucial role in all stages of heart disease, especially in the short-term aftermath of a heart attack. Recent studies have examined different inflammatory markers to determine if higher levels are linked to poorer outcomes after a heart attack. Some research has already found a connection between CRP and myocardial infarction [10, 11, 12]. IL-6 family cytokines have been shown to play a significant role in the various stages of atherosclerosis progression, from the initial inflammation to reperfusion injury, healing, and scar tissue inflammation post-heart attack [13-14]. Research indicates that the levels of pro-inflammatory cytokines IL-6 and CRP in the bloodstream are correlated with the size of a heart attack. Elevated circulating levels of IL-6 have been linked to an increased risk of heart attack, particularly during acute coronary syndromes (ACS) which are associated with a poorer long-term prognosis [15]. Studies have identified IL-6 and hsCRP as strong indicators of cardiovascular events in patients with unstable angina, as well as independent predictors of myocardial infarction or heart attacks. Elevated levels of Interleukin-6 and C-reactive protein can serve as early indicators of the progression of atherogenic risk [16]. High Sensitivity C-reactive protein (HsCRP) has been identified as a reliable biomarker for predicting cardiovascular disease (CVD) risks, particularly in individuals with Type 2 diabetes [17-18]. In addition, increased ratios of lipoproteins along with elevated levels of Hs-CRP may significantly elevate the risk of developing cardiovascular issues [19]. The correlation between rising levels of C-reactive protein and LDL cholesterol has been linked to the development of cardiac abnormalities. It is crucial to highlight the importance of promptly measuring lipid levels, particularly LDL cholesterol, and interleukin-6, as it improves the accuracy of predicting future myocardial infarction and coronary mortality compared to assessing lipids or IL-6 alone [20]. Studies have shown that elevated levels of glycated hemoglobin are major risk factors in the progression of diabetes mellitus, atherosclerosis, myocardial infarction, renal dysfunction, hypertriglyceridemia, and obesity [21]. Furthermore, high levels of HbA1c and dyslipidemia are independent risk factors for CVD in individuals at extremely high risk [22- 23]. Therefore, it is of interest to examine the relationship between serum interleukin-6 (IL-6) and hsCRP as potential predictors of risk factors for myocardial infarction. The infarct size at 72 hours post PCI was predicted only by baseline levels of IL-6 [36].

## Materials and Methods

## Study design and sampling:

We investigated patients who had experienced Myocardial Infarction and were analyzed using a standardized methodology along with a pre-designed form. The study was carried out in the Outpatient Department (OPD) and Inpatient Department (IPD) of Emergency Medicine at Santosh Medical College, Santosh Deemed to be University, Ghaziabad, and Uttar Pradesh, India.

## The criteria for inclusion in the study were as follows:

Patients must be less than 18 years of age, demonstrate elevated levels of CK-MB and TnT, and have experienced chest discomfort lasting more than 24 hours. This discomfort should be indicative of rapid or prolonged myocardial ischemia, lasting more than 20 minutes, or recurring episodes at rest or with light exercise, accompanied by at least one of the following indications: new or suspected ECG changes (such as transient ST-segment elevation over 0.5 mm, ST-segment depression over 0.5 mm, or T-wave inversion over 3 mm in two or more contiguous leads), as well as elevated cardiac markers (CK levels 2 times greater than the upper limit of normal).

## Exclusion criteria:

included individuals over the age of 20, with a heart rate exceeding 120 beats per minute, suffering from septic shock, acute respiratory distress syndrome, severe hepatic failure, significant mechanical obstruction, allergic reactions to the study drug or its components, anemia (hemoglobin under 8 g/dl), or pregnancy.

## Assessment of variables:

The assessment of variables involved measuring high sensitivity C-reactive protein (hsCRP) utilizing the immunoturbidimetry method, and estimating interleukin-6 (IL-6) uses a Sandwich ELISA method kit. Data regarding demographics and socioeconomic factors was obtained through a survey, which included inquiries about age, gender, personal medical history, family medical history, level of education, alcohol consumption, tobacco use, smoking habits, physical fitness routine, and quality of sleep. Smoking habits were categorized as never smoked, former smoker, or current smoker based on individual reports. In this study, participants who identified as current smokers were classified as smokers. Hypertension was defined as having a history of high blood pressure, taking antihypertensive medications, having a systolic blood pressure of 140 mmHg or higher, or a diastolic blood pressure of 90 mmHg or higher. Diabetes mellitus (DM) was defined as self-reported diagnosis, use of insulin or oral hypoglycemic medication, or a fasting blood glucose level of 126mg/dl or greater. Body mass index (BMI) calculations were also performed as part of the study.

## BMI evaluation:

Data on measurements related to body mass index (BMI), such as anthropometric, lifestyle, and dietary information, were collected from surveys conducted among both women and men. Any missing details were filled in using data from previous surveys. The BMI was determined by calculating the weight in kilograms divided by the square of the height in meters. Blood Pressure Monitoring: Blood pressure readings, including systolic and diastolic measurements, were taken while the participants were seated after a 5-minute rest period, using a mercury-based sphygmomanometer.

## Data analysis:

Statistical analysis was carried out using SPSS version 21 software. Continuous variables were presented as mean ± standard deviation, while categorical variables were shown as percentages. These variables were compared using Chi-Squared tests. IL-6 and hs-CRP levels were categorized into quartiles, and hazards ratios were estimated using Cox regression models based on different concentrations of these markers. A statistically significant difference was considered when p value was less than 0.05. Correlation coefficients were calculated between age and logarithmically transformed values, and differences in clinical characteristics among quartiles were compared. Further analyses included the evaluation of overall comparisons using the Mantel-cox method and the computation of all-cause mortality, death from myocardial infarction (MI), and MI-free survival rates based on different levels of biomarkers and other inflammation-sensitive proteins divided into quartiles, using the Kaplan-Meier method with the generalized Wilcoxon rank sum test. The 75th percentile was used as the cut-off point to define membership in the top quartile of another protein(s).

## Results:

**Figure 1:**
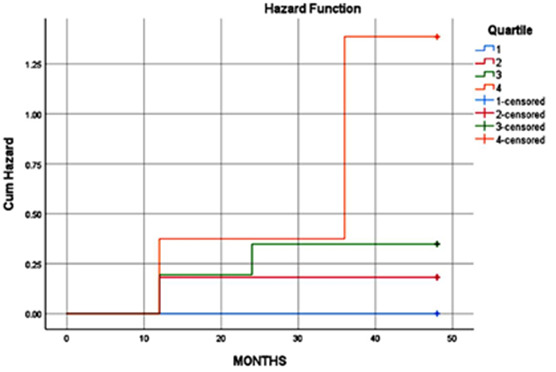
Kaplan-Meier curve for Hazard function analysis for risk factor associated to MI by IL-6 quartile group; analysis was carried out using SPSS version 21 software

**Figure 2:**
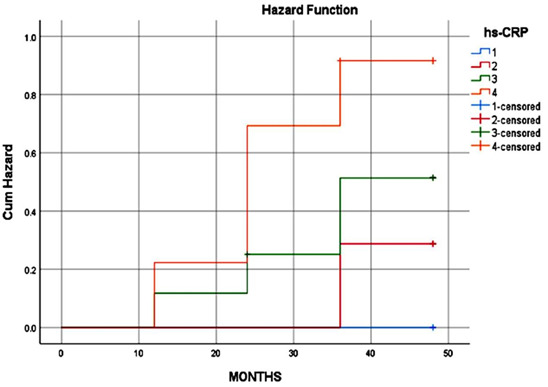
Kaplan-Meier curve for Hazard function analysis for risk factor associated to MI by hs-CRP quartile group; analysis was carried out using SPSS version 21 software

The research involved 50 patients with an average age of 57.86 years. These patients were divided into quartiles based on their IL-6 and hs-CRP concentrations. Table 1 summarizes the demographic characteristics and comorbid illnesses of the patients, while Table 2 outlines the clinical characteristics. The IL-6 quartile divisions were at 10pg/ml, 20pg/ml, and 40pg/ml, while the hs-CRP quartile divisions were at 1mg/ml, 2mg/ml, and 3mg/ml. Various clinical factors like age, BMI, sex, blood sugar fasting (BSF), habits, and physical activity were found to be associated with IL-6 levels. Higher levels of hs-CRP were also linked to more severe clinical conditions. The study revealed a higher proportion of male patients. Additionally, higher hs-CRP quartiles were correlated with an increased risk of cardiovascular factors and other comorbid conditions. The log rank analysis revealed that baseline IL-6 and hs-CRP were both independently associated with an increased risk of myocardial infarction (MI). Participants in the highest quartile (Q4) of hs-CRP had a significantly higher risk of MI compared to those in the lowest quartile (Q1). This finding was further supported by the data presented in Table 3, which included estimated values and standard error values for each quartile at the 25th, 50th, and 75th percentiles. Similar results were seen for IL-6, with participants in the highest quartile also showing a significantly increased risk of MI compared to those in lower quartiles. The events observed were summarized in Table 3, with the highest number of events occurring in the Q4 quartiles of both IL-6 and hs-CRP. Table 4 provided a summary of the log rank values for each biomarker, with the p-values for hs-CRP and IL-6 being greater than 0.005 and 0.005, respectively. Hazard functions for each quartile with respect to time were analyzed, showing a higher risk of MI in the Q4 quartile. Kaplan-Meier plots in Figure 1 and Figure 2 further illustrated the unadjusted associations between quartile groups of IL-6 and hs-CRP and the risk of MI, indicating a higher risk in the Q4 quartile for both biomarkers.

## Discussion:

The comparison of biomarkers to determine the effectiveness of IL-6 and hsCRP in diagnosing myocardial infarction (MI) is of interest. The importance of elevated baseline hsCRP levels in diagnostic procedures is shown. The link between hsCRP and myocardial infarction was found to be significant in male patients [23]. We investigated the independent relationship between IL-6 and hsCRP in relation to myocardial infarction. It was discovered that IL-6 is also associated with an increased risk of myocardial infarction and overall mortality due to heart failure. Additionally, hsCRP has been linked to a higher risk of cardiovascular death. Previous studies have also supported the association between IL-6 and hsCRP as risk factors for myocardial infarction [24].

Data shows that the individual roles of hsCRP and IL-6 in healthy individuals, with evidence of their association with myocardial infarction. Recent research on myocardial infarction patients showed a strong correlation between IL-6 and hsCRP with thin-cap fibro atheroma. Patients with high levels of IL-6 were found to have a significantly increased risk of hospitalization compared to those with high hsCRP levels [25]. Furthermore, IL-6 was identified as an independent predictor of poor outcomes in terms of analyzing cardiovascular risk factors, although not as effective as hsCRP. Our findings suggest that hsCRP is a valuable predictor of myocardial infarction, consistent with previous studies conducted by Biasucci *et al.* [26] which found that hsCRP levels exceeding 3 mg/l were significantly associated with the occurrence of myocardial infarction. Moreover, research by Blangy *et al.* [27] also indicated a higher incidence of myocardial infarction associated with elevated hsCRP levels. Furthermore, Bonny *et al.* [28] observed that high CRP concentrations indicating systemic inflammation were more prevalent following spontaneous ventricular arrhythmia in patients with arrhythmogenic right ventricular cardiomyopathy (ARVC). Lastly, Shehab *et al.* in 2004 highlighted the utility of hsCRP as an indicator of long-term risk of sudden cardiac death in healthy males and those with chronic heart failure [24].

Short-term increase in IL-6 levels is expected as a response to acute myocardial infarction, while sustained production of IL-6 can lead to heart failure [29-30]. Studies have shown that hsCRP serves as a marker of inflammation, promoting the recruitment of monocytes to plaque build-up in arteries. This process can also disrupt the production of nitric oxide, which is essential for healthy endothelial function, ultimately contributing to the development of atherosclerosis [31]. Recent research has highlighted the potential of hs-CRP as a prognostic indicator, particularly in cases of heart attack, stroke, peripheral artery disease, and atrial fibrillation recurrence [32- 33]. The Kaplan-Meier methodology is employed for statistical analysis to approximate the survival function using data on lifetimes. By using the Kaplan-Meier estimator, the likelihood of survival at various time intervals can be computed based on observed data. These visual representations are crucial for comparing and viewing survival patterns among distinct cohorts or interventions. In this investigation, Kaplan-Meier plots delved into examining hazard functions for each quartile over time, with a specific focus on myocardial infarction (MI) risks. It was evident from the analysis that the highest risk of MI was evident in the fourth quartile. Furthermore, the Kaplan-Meier plots visually revealed unadjusted correlations between quartiles of IL-6 and hs-CRP biomarkers and the probability of MI. The plots illustrated a heightened risk of MI in the fourth quartile for both IL-6 and hs-CRP biomarkers. Overall, the outcomes indicate a notable link between elevated levels of these biomarkers and an increased susceptibility to MI [34]. Furthermore, elevated hsCRP levels have been identified as an independent risk factor for various cardiovascular conditions. Some studies have even suggested a link between increased hsCRP levels and the recurrence of atrial fibrillation, which may be attributed to changes in the heart's electrical and structural components, leading to myocardial fibrosis. A study by Sajadieh *et al.* [35] found that individuals with hsCRP levels exceeding 2.5 mg/l were at a significantly higher risk of death or experiencing a heart attack. A correlation was observed between higher hsCRP levels and increased risk of myocardial infarction incidence. Further investigations are needed to elucidate the specific mechanisms underlying myocardial infarction triggered by elevated levels of hsCRP and IL-6 as pro-inflammatory markers.

## Conclusion:

Elevated levels of high-sensitivity C-reactive protein (hsCRP) were found to be a significant biomarker for predicting the likelihood of having a heart attack (myocardial infarction), indicating a link between heart attack risk and inflammation throughout the body. Our study also revealed that the combination of interleukin-6 (IL-6) and hsCRP biomarkers is the most reliable predictor of risk factors for myocardial infarction. However, IL-6 alone was deemed less effective in prediction due to its lower efficiency and sensitivity. Our study strongly suggests that IL-6 could be a powerful marker in evaluating the Myocardial Infarction.

## Author Contributions:

Writing original Draft, Manish Kumar Verma, Virendra Verma, Pavan Kumar Sharma, Shiv Shanker Tripathi and Devajit Sarmah, Writing review and editing, Manish Kumar Verma , Shashi Prabha Singh, Rajni Patel, Poonam Verma Supervision, Manish Kumar Verma, Virendra Verma, Pavan Kumar Sharma, Shashi Prabha Singh, Devajit Sarmah, Investigation, Manish Kumar Verma , Deepa Arya, , Poonam Verma Validation, Manish Kumar Verma, Virendra Verma, Pavan Kumar Sharma, Shiv Shanker Tripathi and Devajit Sarmah All authors have read and agreed to the published version of the manuscript.

## Consent for publication:

All authors have declared that no financial support was received from any organization for the submitted work.

## Funding:

No funding was received for this research.

## Figures and Tables

**Table 1:** Demographic and baseline characteristic of Quartile group of IL-6 and hs-CRP

	**IL-6**					**hs -CRP**				
CHARACTERISTICS	Q1 (≤10) pg/ml	Q2 (11-20) pg/ml	Q3 (21-40) pg/ml	Q4 (≤40) pg/ml	p -Value	Q1 (≤1) mg/l	Q2 (1-2) mg/l	Q3 (2-3) mg/l	Q4 (≥3) mg/l	p -Value
NO. OF PATIENTS (n)	5	12	17	16		4	8	18	20	
AGE (YEARS)	59±1.30	61±1.91	62±1.03	49±1.32	<0.001	60±1.92	62±0.09	59±1.13	65±1.93	<0.001
MALE n (%)	4 (80)	9 (75)	16(94.11)	14 (87.5)	<0.001	4 (100)	8 (100)	13 (72.2)	17 (85)	<0.001
FEMALE n (%)	1(20)	3 (25)	1(5.88)	2 (12.5)	<0.001	0(0)	1 (12.5)	3 (16.6)	2 (10)	<0.001
BSF (mg/dl)	129	141	164	212		120	148	197	209	
BMI (kg/m2)	22.2±2.42	25.19±1.96	30.3±3.36	27.21±3.13	<0.001	23.1±2.10	24.1±1.16	29.1±3.13	28.2±2.19	<0.001
HYPERTENSION n (%)	2 (40)	9 (75)	12 (70.58)	14 (87.5)	<0.001	0(0)	2 (25)	8 (44.4)	5 (25)	<0.001
DIABETES n (%)	1(20)	5(41.6)	8 (47)	9 (56.2)	<0.001	0(0)	6 (75)	9 (50)	14 (70)	<0.001
DYSLIPIDEMIA n (%)	2(40)	8 (66.6)	10 (58.8)	15 (93.7)	<0.001	1 (25)	7 (87.5)	11 (61.11)	18 (90)	<0.001
ALCOHOL n (%)	3(60)	7 (58.3)	9 (52.9)	14 (87.5)	<0.001	2(50)	1 (12.5)	14 (77.7)	12 (60)	<0.001
TOBACCO n (%)	3(60)	10 (83.33)	11 (64.7)	11 (68.7)	<0.001	1 (25)	3 (37.5)	13 (72.2)	9 (45)	<0.001
SMOKING n (%)	3(60)	8(66.6)	12(70.5)	14 (87.5)	<0.001	1 (25)	3 (37.5)	4 (22.2)	6 (30)	<0.001
PHY. ACTIVITY n (%)	1(20)	0 (0)	0 (0)	0 (0)	<0.001	0(0)	0 (0)	0 (0)	0 (0)	<0.001
SWEATING n (%)	0(0)	0 (0)	0 (0)	0 (0)	<0.001	0(0)	0(0)	0(0)	0 (0)	<0.001
SHORTNESS OF BREATH n (%)	0(0)	1(8.33)	3(17.6)	8 (50)	<0.001	0(0)	0 (0)	1 (5.55)	2 (10)	<0.001
VOMITING n (%)	0(0)	0 (0)	0 (0)	0 (0)	<0.001	0(0)	0 (0)	0 (0)	0 (0)	<0.001
COUGH n (%)	0(0)	0 (0)	0 (0)	0 (0)	<0.001	0(0)	0(0)	0(0)	0 (0)	<0.001
PALPITATION n (%)	0 (0)	0 (0)	0 (0)	1 (6.25)	<0.001	0 (0)	0 (0)	0 (0)	0 (0)	<0.001
DIZZINESS n (%)	0(0)	0 (0)	1 (5.88)	0 (0)	<0.001	0(0)	0(0)	0 (0)	0 (0)	<0.001
ABDOMINAL PAIN n (%)	0(0)	0 (0)	0 (0)	0 (0)	<0.001	0(0)	0(0)	0 (0)	0 (0)	<0.001
CHEAST PAIN STEMI n (%)	5(100)	6 (50)	5 (29.4)	7 (43.7)	<0.001	2 (50)	2 (25)	1 (5.55)	2 (10)	<0.001
CHEAST PAIN NSTEMI n (%)	0(0)	0 (0)	1 (5.88)	1 (6.25)	<0.001	0(0)	0(0)	0 (0)	0 (0)	<0.001
PATHOLOGICAL Q WAVE n (%)	0(0)	0(0)	0 (0)	0 (0)	<0.001	0(0)	0(0)	0 (0)	0 (0)	<0.001
ANTERIOR WALL n (%)	4(80)	8 (66.6)	9 (52.9)	9 (56.2)	<0.001	1 (25)	4 (50)	2 (11.1)	2 (10)	<0.001
INFERIOR WALL n (%)	2(40)	4 (33.3)	6 (35.29)	7 (43.7)	<0.001	1 (25)	1 (12.5)	1(5.55)	3 (15)	<0.001
A+I WALL n (%)	0(0)	0 (0)	0 (0)	1 (6.25)	<0.001	0(0)	0(0)	1 (5.55)	0 (0)	<0.001
ANTERIOR+ LATERAL WALL n (%)	0(0)	0 (0)	0(0)	1 (6.25)	<0.001	0(0)	0(0)	0 (0)	0(0)	<0.001
INFEROR + LATERAL WALL n (%)	0(0)	0 (0)	0 (0)	1 (6.25)	<0.001	0(0)	0(0)	1 (5.55)	0(0)	<0.001
NORMAL SINUS RHYTHAM n (%)	3(60)	5(41.6)	7 (41.17)	1 (6.25)	<0.001	2 (50)	4 (50)	3 (16.6)	0 (0)	<0.001
SINUS TACHYCARDIA n (%)	0(0)	0(0)	1 (5.88)	1 (6.25)	<0.001	0(0)	0 (0)	1 (5.55)	0 (0)	<0.001
SINUS BRADYCARDIA n (%)	0(0)	0 (0)	0(0)	1 (6.25)	<0.001	0(0)	0 (0)	0 (0)	0 (0)	<0.001
VENTRICULAR TACHYCARDIA n (%)	0(0)	0 (0)	1 (5.88)	0(0)	<0.001	0(0)	0 (0)	0 (0)	0 (0)	<0.001
AV BLOCK n (%)	0(0)	0(0)	0 (0)	1 (6.25)	<0.001	0(0)	0(0)	0(0)	1 (5)	<0.001

**Table 2:** Baseline clinical observation of IL-6 and hs-CRP

**CHARACTERISTICS**	**IL-6**					**hs -CRP**				
	**Q1 (≤10)pg/ml**	**Q2 (11-20)pg/ml**	**Q3(21-40)pg/ml**	**Q4 (≤40)pg/ml**	**P- VALUE**	**Q1(≤1)mg/l**	**Q2(1-2)mg/l**	**Q3(2-3)mg/l**	**Q4(mg/l)≥3**	**p -Value**
**NO. OF PATIENTS (n)**	**5**	**12**	**17**	**16**		**4**	8	**18**	**20**	
TC	128±1.16	153±1.03	129±2.31	132±2.33	<0.001	127±0.75	151±1.32	128±1.78	132±1.31	<0.001
TG	79±1.58	82±1.66	81±1.36	88±1.10	<0.001	77±0.39	79±1.32	81±0.61	87±132	<0.001
HDL	54±0.03	64±1.31	82±1.42	98±0.75	<0.001	53±0.09	62±1.93	81±1.31	97±0.32	<0.001
LDL	60±1.17	73±1.47	77±0.04	92±1.45	0.201	61±1.52	72±1.02	76±1.36	91±1.01	0.061
VLDL	21.9±0.39	24.6±1.25	30.1±0.08	30.7±1.42	<0.001	22.4±0.13	23.1±1.10	31.1±0.09	29.3±2.19	<0.001
TG/HDL-c	4.21±1.19	3.05±0.91	5.66±1.01	9.36±0.40	<0.001	4.72±0.63	3.47±1.10	4.21±1.81	5.31±0.12	<0.001
TC/HDL-c	4.73±0.73	3.57±1.42	4.31±1.96	5.10±1.40	<0.001	23.1±2.10	24.1±1.16	29.1±3.13	28.2±3.16	<0.001

**Table 3:** Percentile observed for biomarker's quartile (Estimated values and std. Error)

**Percentile**	**IL-6**	**Q1**	**Q2**	**Q3**	**Q4**	**Overall**
25%	Estimated	....	....	....	36	....
	Std.Error	....	....	....	....	....
50%	Estimated	....	....	....	36	....
	Std. error	....	....	....	5.938	....
75%	Estimated	....	....	24	12	36
	Std. error	....	....	....	....	9.153
	hs-CRP	Q1	Q2	Q3	Q4	Overall
25%	Estimated	....	....	....	....	....
	Std. error	....	....	....	....	....
50%	Estimated	....	....	....	24	....
	Std. error	....	....	....	0.703	....
75%	Estimated	....	36	36	24	24
	Std. Error	....	....	9.752	4.472	5.013

**Table 4:** Case progressing summery of participants observed via Kaplan-Meier method

**IL-6**		**Q1**	**Q2**	**Q3**	**Q4**	**Overall**
TOTAL NO.		5	12	17	16	50
NO. OF EVENTS		0	2	5	12	19
CENSORED	NUMBER	5	10	12	4	31
	PERCENTAGE	100%	83.30%	70.60%	25.00%	62.00%
Hs-CRP		Q1	Q2	Q3	Q4	Overall
TOTAL NO.		4	8	18	20	50
NO. OF EVENTS		0	2	7	12	21
CENSORED	NUMBER	4	6	11	8	29
	PERCENTAGE	100%	75.00%	61.10%	40.00%	58.00%

**Table 5:** Overall comparison of biomarkers (Log rank)

**BIOMARKERS**	**CHARACTERISTICS**	**CHI-SQUARE**	**Sig df**
Log Rank	IL-6	12.807	0.005
	hs-CRP	7.247	0.064
